# The Anti-Tumor Efficacy of Verbascoside on Ovarian Cancer *via* Facilitating CCN1-AKT/NF-κB Pathway-Mediated M1 Macrophage Polarization

**DOI:** 10.3389/fonc.2022.901922

**Published:** 2022-06-17

**Authors:** Yu Ren, Jinying He, Wenhua Zhao, Yuzhen Ma

**Affiliations:** ^1^ Scientific Research Department, Inner Mongolia People’s Hospital, Hohhot, China; ^2^ Reproductive Medicine Centre, Inner Mongolia People’s Hospital, Hohhot, China

**Keywords:** verbascoside, ovarian cancer, M1 macrophage polarization, CCN1, Akt/NF-κB pathway

## Abstract

**Background:**

Ovarian cancer (OC) is the leading cause of gynecological cancer-related mortality. Verbascoside (VB) is a phenylpropanoid glycoside from Chinese herbs, with anti-tumour activities. This study aimed to investigate the effects and mechanism of VB on OC.

**Methods:**

OC cell lines SKOV3 and A2780 were used in this study. Cell viability, proliferation, and migration were measured using CCK-8, clonogenic, and transwell assays, respectively. Apoptosis and M1/M2 macrophages were detected using flow cytometry. The interaction between VB and CCN1 was predicted by molecular docking. The mRNA expression of CCN1 was detected by RT-qPCR. The protein levels of CCN1, AKT, p-AKT, p65, and p-p65 were determined by western blotting. A xenograft mice model was established for *in vivo* validation.

**Results:**

VB inhibited OC cell proliferation and migration in a dose-dependent manner, and promoted apoptosis and M1 macrophage polarization. VB downregulated CCN1 and inhibited the AKT/NF-κB pathway. LY294002, an AKT inhibitor, potentiated the anti-tumour effects of VB. CCN1 overexpression weakened the anti-tumour effects of VB and VB + LY294002. *In vivo* experiments verified that VB inhibited tumour growth and promoted M1 polarization, which is regulated by the CCN1-mediated AKT/NF-κB pathway.

**Conclusion:**

VB triggers the CCN1-AKT/NF-κB pathway-mediated M1 macrophage polarization for protecting against OC.

## Introduction

Ovarian cancer (OC) is one of the most lethal tumours in the female reproductive tract (1). OC generally has no particular symptoms or clinical features at an early stage, making it difficult to identify, and 70–80% of patients are diagnosed at an advanced stage (2, 3). At present, the major therapy for OC is surgery combined with chemotherapy, including platinum and paclitaxel (4). However, malignant tumours may recur at distant sites or at primary stage following treatment (5, 6). Therefore, the identification of effective drugs for OC treatment is urgently required.

Traditional Chinese medicine (TCM) is an important feature of Chinese national health policies. TCMs tend to have beneficial therapeutic efficacy with minimal side effects, thereby preventing and treating various diseases (7). Verbascoside (VB) is a phenylpropanoid glycoside that exists in various herbal extracts. VB has recently received considerable attention owing to its significant clinical value, including antioxidative, anti-inflammatory, antibacterial, and antifungal properties (8–12). Moreover, VB also possesses anti-tumour activity against various cancers, such as glioblastoma, prostate cancer, and breast cancer (13–17). For example, VB has an anti-tumour role in breast cancer by suppressing cell proliferation (16). VB can effectively inhibit cell growth and migration, and promote cell apoptosis and autophagy in glioblastoma (15). VB also mitigates the cell proliferation and aggressiveness in prostate cancer (17). However, the efficacies and potential mechanisms of VB against OC remain unclear.

Cellular communication network factor 1 (CCN1), also named cysteine-rich angiogenic inducer 61 (CYR61), is a matricellular protein of the CCN family. Aberrant CCN1 expression is associated with various cancers, such as gastric and mammary cancers, by regulating cell adhesion, proliferation, migration, and differentiation (18–20). Accumulating evidence indicates that CCN1 is associated with the development of OC. Shi et al. reported that CCN1 has high expression in the advanced stage of OC and may promote tumour metastasis and progression (21). Shen et al. indicated that CCN1 may be an effective biomarker for the poor prognosis of OC (22). Furthermore, The protein kinase B (AKT)/nuclear factor-κB (NF-κB) signalling pathway acts as a pivotal role in regulating cell proliferation, migration, and apoptosis in cancers (23–26). A previous study confirmed that CCN1 positively regulates tumour growth and helps cells resist apoptosis *via* activating the AKT/NF-κB signalling pathway in pancreatic cancer (27). We speculated that VB may exert an anti-tumour effect by affecting the CCN1-mediated AKT/NF-κB signalling pathway in OC. Moreover, numerous evidences have indicated that AKT/NF-κB pathway exerts a pivotal role in tumour-associated macrophage polarization (28, 29). Tumour-associated macrophages are an essential component of the tumour microenvironment and can be polarized into M1 and M2 types (30). Wang et al. suggested that microRNA-155-5p promotes M2 macrophage polarization by activating the AKT/NF-κB pathway in pancreatic cancer (29). Le et al. reported that triptolide can inhibit the cell invasion and migration in OC by suppressing M2 polarization, which is regulated by the AKT/NF-κB pathway (28). In addition, it has been reported that CCN1 can facilitate macrophage migration and infiltration in tumor tissues (20). A previous study indicated that VB possesses the effect on inhibiting macrophage infiltration (31). However, whether macrophage polarization regulated by the CCN1-mediated AKT/NF-κB signalling pathway is associated with the anti-tumour effect of VB remains illusively.

In this article, the anti-tumour effect of VB was evaluated by determining the proliferation, migration, and apoptosis of OC cells. The underlying mechanism of VB on OC development and macrophage polarization involving in the CCN1-mediated AKT/NF-κB pathway were also determined. This study may identify an effective drug for OC treatment and offer promising prospects for exploring the potential mechanisms of action of VB against OC.

## Materials and Methods

### Cell Culture and Treatment

Human OC cell lines (SKOV3 and A2780) were obtained from the Cell Bank of the Chinese Academy of Sciences (Shanghai, China). SKOV3 and A2780 cells are the most commonly used OC cells in *in vitro* therapeutic studies. SKOV3 cells present epithelial morphology and originated from tumor tissue, whereas A2780 cells with round morphology from ascites. Using cell lines with different morphology and origin in this study can better confirm the therapeutic effect of VB on OC (32). SKOV3 and A2780 cells were maintained in Roswell Park Memorial Institute (RPMI)-1640 medium (Gibco, NY, USA) with 10% fetal bovine serum (FBS; Gibco) and 1% penicillin-streptomycin in an incubator at 37°C containing 5% CO_2_/95% air. For subsequent experiments, SKOV3 and A2780 cells were treated with different VB concentrations (5, 10, 20, 40, and 80 µM) and/or 20 µM LY294002. The concentrations of VB used in this study were decided according to previous studies (17, 33, 34). LY294002 is an AKT inhibitor that was used to block AKT/NF-κB pathway in this study (35).

### Cell Transfection

SKOV3 and A2780 cells were cultured into six-well plates (1 × 10^4^ cells/well) at 37°C until reaching 80% confluence. Lentivirus-based vectors containing CCN1 (lenti-CCN1) or negative control (lenti-NC) were purchased from Inovogen (Beijing, China). Lenti-CCN1 or lenti-NC were added into cell plates together with the Lipofectamine 3000 reagents (Thermo Fisher Scientific, MA, USA) as recommended by the suppliers. After 48 h post-transfection, cells were treated with 20 µM VB and/or 20 µM LY294002.

### Cell Counting Kit 8 (CCK8) Assay

The viability of SKOV3 and A2780 cells was measured by a CCK8 assay. CCK8 is a convenient assay by utilizing the highly water-soluble tetrazolium salt WST-8 that produces a water-soluble formazan dye upon reduction in the presence of an electron carrier, thereby determining the number of viable cells in cell proliferation using the sensitive colorimetric assay. Briefly, resuspended cells were cultured in a 96-well plate (1 × 10^4^ cells/well) for 24, 48, and 72 h. Next, cells were incubated with 10 µL of CCK8 solution (Beyotime, Jiangsu, China) for 2 h at 37°C with 5% CO_2_. Subsequently, the absorbance was detected under a wavelength of 450 nm using a DR-200Bs microplate reader (Diatek, Jiangsu, China). Meanwhile, half maximal inhibitory (IC50) concentrations of VB treating SKOV3 and A2780 cells at 48 h were calculated.

### Clonogenic Assay

Cells were cultured into a 12-well plate (200 cells/well) for 24 h at 37°C with 5% CO_2_. After culturing for 1 week, cells were fixed with 10% precooled methanol for 20 min at 4°C, and then stained with 1% crystal violet (Solarbio, Beijing, China) for 10 min. Colonies were photographed using a digital SLR camera (Nikon, Tokyo, Japan) and counted under a light microscope (Olympus, Tokyo, Japan).

### Transwell Migration Assay

Cells were resuspended to 1 × 10^5^ cells/mL with serum-free medium and then added into the upper Transwell chamber (Corning, NY, USA) at 200 µL/well. Simultaneously, 500 µL medium with 10% FBS was seeded into the lower chamber. After culturing for 24 h at 37°C, the cells were immobilized with 4% polyformaldehyde at 37°C for 5 min and then stained with 0.1% crystal violet at room temperature for 15 min. Then, cells were photographed and counted under a light microscope (Olympus). Cell migration was quantified by counting stained cells in randomly-selected five fields (20×) and calculating the average.

### Cell Apoptosis Assay

The apoptosis of SKOV3 and A2780 cells was detected by flow cytometry after Annexin V-FITC/propidium iodide (PI) staining (Beyotime). Briefly, cells (1 × 10^5^ cells/mL) were incubated with Annexin V-FITC for 15 min at 4°C in a dark box. Next, PI was added to the mixture and reacted for 15 min in the dark. The apoptosis ratio was determined using a BD FACScanto II flow cytometer (BD Biosciences, CA, USA).

### Macrophage Polarization and Identification

THP-1 monocytes were purchased from the Cell Bank of the Chinese Academy of Sciences (Shanghai, China) and selected for the investigation of macrophage polarization based on the previous studies (36, 37). THP-1 monocytes were cultured in RPMI-1640 medium with 10% FBS, 1% antibiotic-antimycotic, and 1% sodium pyruvate. For macrophage polarization, cells were pretreated with 1 µg/mL phorbol myristate acetate (PMA) overnight. THP-1 monocytes were polarized into M1 type by treatment with 100 ng/mL of lipopolysaccharide (LPS) and 20 ng/mL of interferon gamma (IFN-γ), and into M2 type by treatment with 20 ng/mL of interleukin (IL)-4 and IL-13 for 24 h. The polarized macrophages were incubated with antibodies CD86-APC and CD206-APC (eBioscience, CA, USA), and then detected using a FACSCalibur flow cytometer (BD Biosciences) with Paint-A-Gate (BD Biosciences).

### Molecular Docking

The chemical structure of VB was downloaded from ZINC database (38). The 3D structure of CCN1 was retrieved from the Protein Data Base (PDB, https://www.rcsb.org/) (39). Molecular docking was conducted using the AutoDock Vina (v1.1.2) (40). During the procedure of molecular docking, water molecules were eliminated and polar hydrogens were added to the protein structure to simulate the intermolecular interactions between CCN1 protein and the ligand in the active site.

### Quantitative Real-Time PCR (RT-qPCR)

Total RNA of OC cells and tissues was extracted using TRIzol reagent (Invitrogen, CA, USA). Thereafter, cDNA was synthesized using a PrimeScript RT reagent kit (Tiangen, Beijing, China). RT-qPCR was performed on a 7500 Real Time PCR System (Applied Biosystems, MA, USA) with a program of 95°C for 3 min, and 40 cycles with 95°C for 12 s and 62°C for 40 s. Primers were showed in **Table 1**. Relative gene expression was calculated using the 2^−ΔΔCt^. GAPDH was used as an internal control.

**Table 1:** Primer sequences used in qRT-PCR.

Genes	Sequences of primers
IL-6	Forward: 5′-AGT CCT GAT CCA GTT CCT GC-3′ Reverse: 5′-CTA CAT TTG CCG AAG AGC CC-3′
CXCL10	Forward: 5′-GTG GCA TTC AAG GAG TAC CTC-3′ Reverse: 5′-TGA TGG CCT TCG ATT CTG GAT T-3′
FN1	Forward: 5′-ACA AGC ATG TCT CTC TGC CA-3′ Reverse: 5′-TTT GCA TCT TGG TTG GCT GC-3′
CCL22	Forward: 5′-ATC GCC TAC AGA CTG CAC TC-3′ Reverse: 5′-GAC GGT AAC GGA CGT AAT CAC-3′
CCN1	Forward: 5′-AAC CCG GAT TTG TGA GGT GC-3′ Reverse: 5′-GCA GGA ACC GCA GTA CTT GG-3′
GAPDH	Forward: 5′-TGT GGG CAT CAA TGG ATT TGG-3′ Reverse: 5′-ACA CCA TGT ATT CCG GGT CAA T-3′

### Western Blotting

Western blot assay was performed according to a previously report (41). OC cells and tissues were lysed in PIPA buffer for extracting total protein. The protein concentration was detected using a BCA protein assay kit (Thermo Fisher Scientific, CA, USA). Thereafter, proteins were separated on 8% SDS-PAGE and then transferred onto polyvinylidene difluoride membranes. Membranes were blocked with 5% skim milk for 1 h and incubated with the primary antibodies against IL-6, CXCL10, FN1, CCL22, CCN1, AKT, p-AKT, p65, and p-p65 (1:1000, Abcam, UK) overnight at 4°C. Followed by that, membranes were incubated with horseradish peroxidase (HRP)-conjugated secondary antibody (1:1000, Abcam, UK) at 37°C for 1 h. Protein bands were visualized using a ECL substrate reagent kit (GE Healthcare, USA) and then photographed on a Gel Doc XR imaging system (Bio-Rad, CA, USA).

### Tumour Xenograft Experiment

Six-week-old female C57BL/6 mice (Chinese Academy of Sciences, Shanghai, China) were subcutaneously implanted with A2780 cells transfected with lenti-NC or lenti-CCN1 (5 × 10^6^ cells/mL). After one week, the mice were intraperitoneally injected with 80 mg/kg/day of VB. The *in vivo* concentration of VB was determined according to previous studies (42–44). Three weeks later, after intraperitoneal injection of 50 mg/kg pentobarbital sodium, the mice sacrificed were killed by cervical dislocation. Tumours from the mice were surgically removed and weighed. Animal experiments have been approved by the Animal and Medical Committee of Inner Mongolia People’s Hospital.

### TUNEL Apoptosis Assay

Tumour tissues from tumour xenograft model mice were fixed in paraffin and sliced into 3 µm thick sections. Apoptosis in tumour tissues was determined using the TUNEL detection kit (Roche, Basel, Germany). Briefly, sections were pre-treated with 0.1% Triton X-100 (Beyotime) and cultured in a TUNEL reaction mixture with PI. Images were captured using a fluorescent microscope (Olympus).

### Immunohistochemistry

The expression of Ki-67 and CCN1 in tumour tissues was detected according to the previously reported immunohistochemical method (45). In brief, paraffin-embedded tumor sections were dewaxed with xylene and dehydrated with graded ethanol. The endogenous peroxidase activity in tissue sections was blocked by immersing in 3% hydrogen peroxide for 10 min and antigen recovery by autoclaving in 0.01 M citrate buffer (pH 6.0) for 30 min. Then, sections were incubated with Ki-67 and CCN1 antibody (1:200) at 4°C overnight, followed by incubation with a secondary antibody (1:1000) at 37°C for 2 h. After incubation, sections were stained with diaminobenzidine and counterstained with Mayer’s hematoxylin. The stained sections were captured by a light microscope (Olympus, Japan).

### Statistical Analysis

Each experiment was repeated thrice independently. All data were represented with mean ± standard deviation and analysed using SPSS 27.0 (IBM, NY, USA). The significant differences among different groups were evaluated using one-way ANOVA, followed by the Tukey’s test. Significant differences were set at *P* < 0.05.

## Results

### VB Inhibits the Malignant Progression of OC Cells

**Figure 1:**
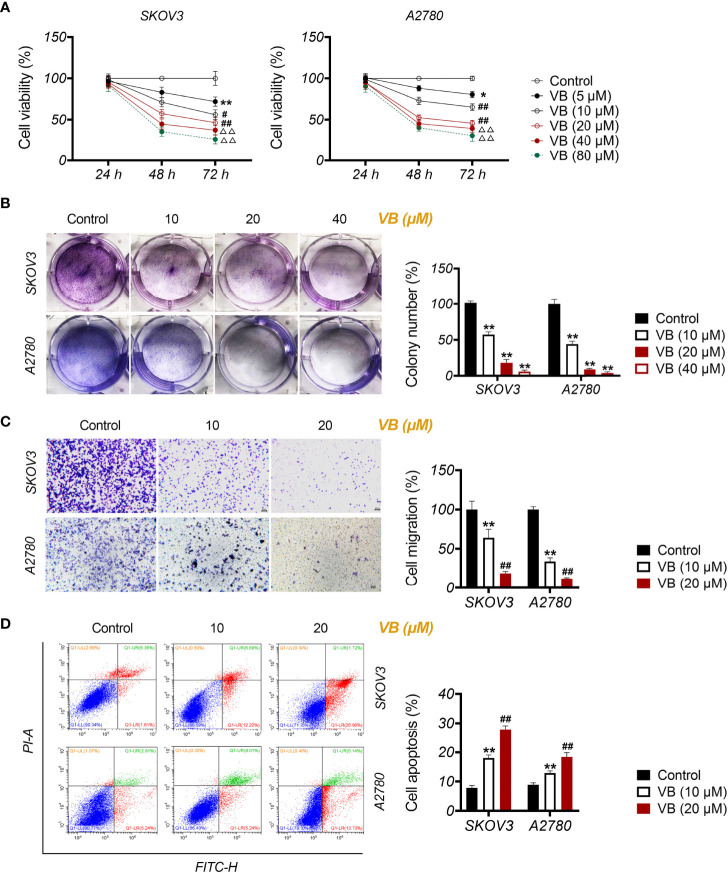
Verbascoside (VB) inhibits proliferation and migration, and promotes apoptosis of ovarian cancer (OC) cells. **(A)** The viability of A2780 and SKOV3 cells was examined using the CCK8 assay. ^*^ *P* < 0.05 and ^**^ *P* < 0.01 vs. the control; ^#^ *P* < 0.05 and ^##^ *P* < 0.01 vs. the VB (5 µM) group; ^ΔΔ^ *P* < 0.001 vs. the VB (10 µM) group. **(B)** The proliferation of A2780 and SKOV3 cells was detected by colony formation assay. **(C)** The migration of A2780 and SKOV3 cells was measured by transwell assay. Scar bar = 50 µm. **(D)** The apoptosis of A2780 and SKOV3 cells was detected by flow cytometry. A2780 and SKOV3 cells were treated with different concentrations of VB (5–80 µM). Each treatment was thrice replicated. Error bars represent the mean ± standard deviation (SD); ^**^ *P* < 0.01 vs. the control; ^##^ *P* < 0.01 vs. the VB (10 µM) group.

To investigate the effect of VB on OC, the viability of OC cell lines (SKOV3 and A2780) was assessed after treatment with increasing VB concentrations (from 5 to 80 μM). **Figure 1A** shows that the viability of SKOV3 and A2780 cells significantly decreases after VB treatment for 48 and 72 h, particularly at high concentrations (*P* < 0.05). The treatments of 10, 20, and 40 µM VB for 24 h were selected for further experiments by eliminating the lowest (5 µM) and highest dose (80 µM). Colony formation revealed that VB significantly suppressed SKOV3 and A2780 cell proliferation in a dose-dependent manner (*P* < 0.01). The IC50 values of VB treating SKOV3 and A2780 cells were 31.28 µM and 28.7 µM, respectively (**Supplementary Figure 1**). OC cell proliferation was limited considerably by 40 μM VB, therefore, 10 and 20 μM VB were used for subsequent experiments (**Figure 1B**). Transwell assays showed that VB inhibited SKOV3 and A2780 cell migration compared to the control (*P* < 0.01; **Figure 1C**). In contrast, the number of apoptotic cells in SKOV3 and A2780 cells was significantly increased following VB treatment compared to that in control cells (*P* < 0.01; **Figure 1D**). The effects of 20 μM VB were more effective in inhibiting the migration and promoting the apoptosis of SKOV3 and A2780 cells than that of 10 μM VB (*P* < 0.01; **Figures 1C, D**).

### VB Suppresses the OC Cell Proliferation by Inducing M1 Polarization of Macrophages

**Figure 2:**
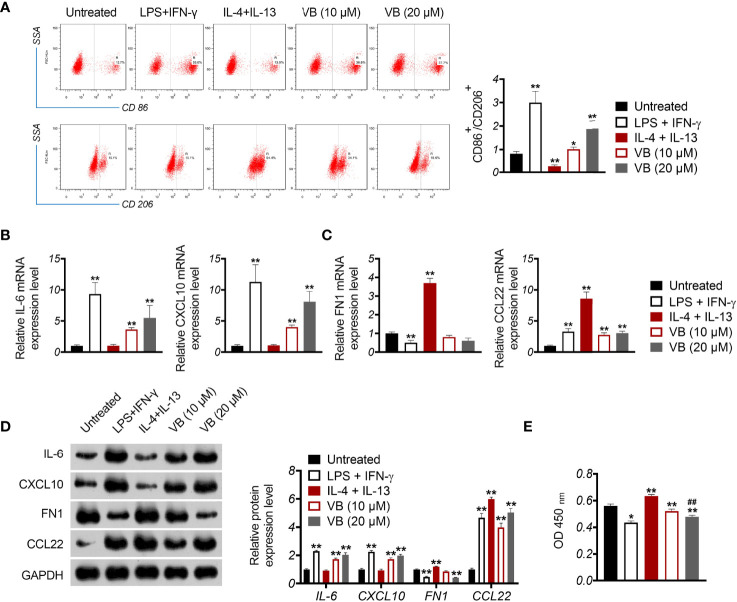
VB promotes M1 macrophage polarization in OC cells. **(A)** The CD86^+^ and CD206^+^ ratio in macrophages were measured by flow cytometry. **(B, C)** The relative mRNA expression levels of IL6, CXCL10, FN1, and CCL22 were examined by RT-qPCR. **(D)** The relative protein expression of IL6, CXCL10, FN1, and CCL22 was measured by Western blotting. **(E)** The proliferation of A2780 cells was detected by CCK8 assay. A2780 cells were co-cultured with THP-1 macrophage and treated with LPS + IFN-γ, IL-4 + IL-13, 10 µM VB, or 20 µM VB. Each trial was replicated at least three times. Data are expressed as the mean ± SD; ^*^ *P* < 0.05 and ^**^ *P* < 0.01 vs. the untreated group; ^##^ *P* < 0.01 vs. the VB (10 µM) group.

To investigate the influence of VB on macrophage polarization, M0 macrophages were treated with LPS + IFN-γ for M1 polarization and treated with IL-4 + IL-13 for M2 polarization. CD86^+^ and CD206^+^ are biomarkers of M1-like and M2-like macrophages, respectively. As expected, treatment with LPS + IFN-γ resulted in increased CD86^+^/CD206^+^ ratio, whereas IL-4 + IL-13 had the opposite effect in comparison to the untreated group (*P* < 0.01; **Figure 2A**). Notably, treatment with VB resulted in a significant increase in CD86^+^/CD206^+^ ratio compared to that of the untreated group (*P* < 0.05; **Figure 2A**). Next, we assessed the expression of M1-associated cytokines (IL-6 and CXCL10) and M2-associated cytokines (FN1 and CCL22). Similar to LPS + IFN-γ, VB treatment increased the expression of IL-6 and CXCL10 in macrophages compared to that of the untreated group (*P* < 0.01; **Figures 2B, D**). The levels of FN1 and CCL22 were no difference and upregulated in macrophages following VB treatment compared to the untreated group, respectively (*P* < 0.01; **Figures 2C, D**). Furthermore, VB inhibited the proliferation of A2780 cells that were co-cultured with LPS + IFN-γ-treated THP-1 macrophage compared with the untreated group (*P* < 0.05; **Figure 2E**).

### VB Suppresses the Malignant Progression of OC Cells Through CCN1-Mediated AKT/NF-κB Signalling Pathway

**Figure 3:**
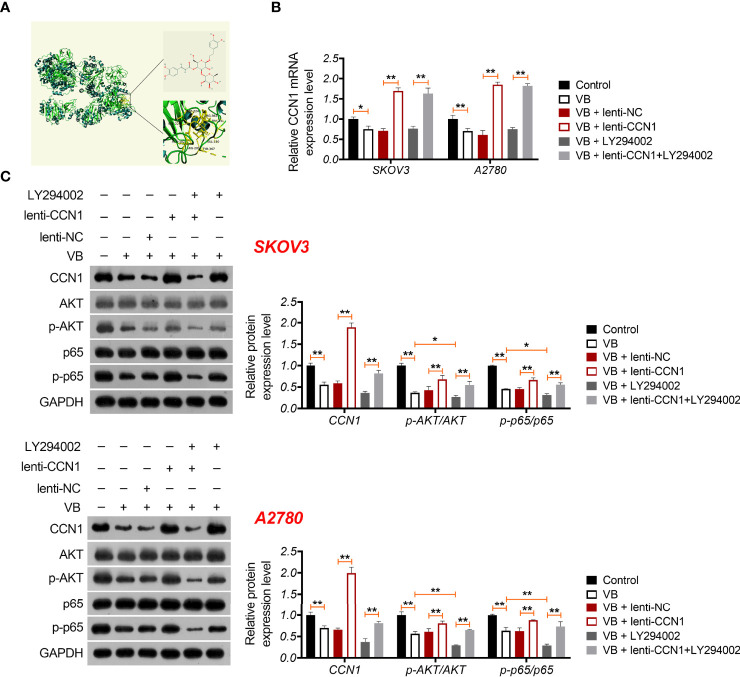
VB interacts with CCN1 and inhibits CCN1-mediated AKT/NF-κB pathway. **(A)** Molecular docking of VB and CCN1. **(B)** The relative mRNA expression of CCN1 was determined by RT-qPCR. **(C)** The protein expression levels of CCN1, AKT, p-AKT, p65, and p-p65 were detected by western blotting in A2780 and SKOV3 cells. Each treatment was replicated three times. Error bars represent the mean ± SD; ^*^ *P* < 0.05 and ^**^ *P* < 0.01.

**Figure 4:**
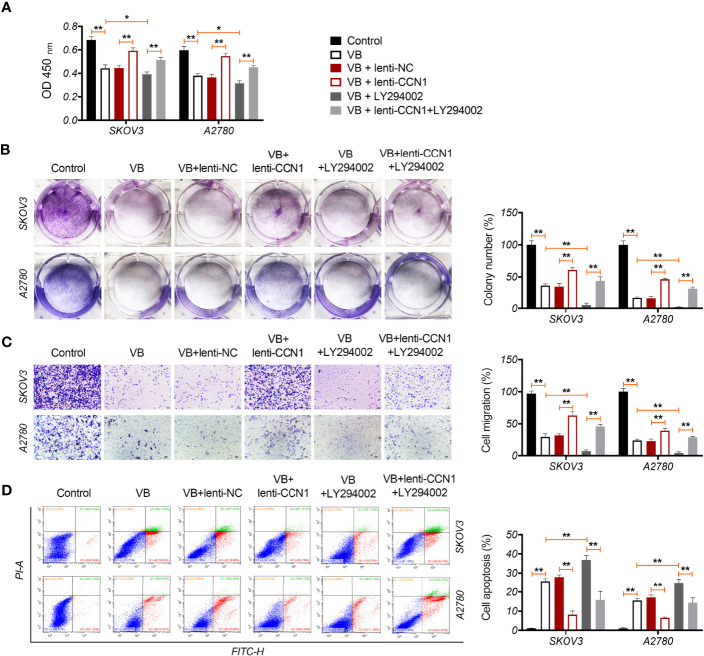
VB suppresses the malignant progression of OC cells *via* CCN1-mediated AKT/NF-κB pathway. **(A)** The viability of A2780 and SKOV3 cells was measured by CCK8 assay. **(B)** The proliferation of A2780 and SKOV3 cells was detected by colony formation assays. **(C)** The migration of A2780 and SKOV3 cells was detected by transwell assay. Scar bar = 50 µm. **(D)** The apoptosis of A2780 and SKOV3 cells was measured by flow cytometry. Each treatment was replicated three times. Error bars represent the mean ± SD; ^*^ *P* < 0.05 and ^**^ *P* < 0.01.

CCN1 is an upregulated gene in the epithelial OC microenvironment (21), and its interaction with VB and CCN1 was confirmed through molecular docking. As shown in **Figure 3A**, VB binds to eight amino acid sites of CCN1, namely ASP-224, ARG-362, TRP-331, VAL-330, TYR-367, ARG-201, ASN-102, and SER-225. To verify the regulatory association between VB, CCN1, and the AKT/NF-κB pathway, the expression of CCN1 and AKT/NF-κB pathway-related proteins (AKT, phospho-AKT, p65, and phospho-p65) was determined. The results showed that VB significantly downregulated both the mRNA and protein levels of CCN1 in SKOV3 and A2780 cells compared with that of the control (*P* < 0.01; **Figures 3B, C**). Simultaneously, VB treatment downregulated the protein level of phospho-AKT (p-AKT) and phospho-p65 (p-p65) in SKOV3 and A2780 cells compared to that of the control (*P* < 0.001; **Figure 3C**). CCN1 overexpression dramatically weakened the effect of VB on inhibiting the expression of CCN1, p-AKT, and p-p65, whereas LY294002 addition enhanced the effect of VB (*P* < 0.05; **Figures 3B, C**). Meanwhile, CCN1 overexpression reversed the effect of VB + LY294002 on inhibiting the expression of CCN1, p-AKT, and p-p65 (*P* < 0.01; **Figures 3B, C**). We further explored whether VB inhibited OC cell progression by regulating the CCN1-mediated AKT/NF-κB pathway. As shown in **Figures 4A–C**, CCN1 weakens the effects of VB on suppressing the viability, proliferation, and migration of SKOV3 and A2780 cells, whereas LY294002 (an AKT inhibitor) potentiates the effects of VB (*P* < 0.05). Similarly, the apoptosis of A2780 and SKOV3 cells promoted by VB was further decreased by CCN1, while it was increased by LY294002 addition (*P* < 0.01; **Figure 4D**). Notably, CCN1 reversed the suppressive effects of VB + LY294002 on the malignant characteristics of A2780 and SKOV3 cells (*P* < 0.01; **Figures 4A–D**).

### VB Promotes M1 Polarization Through Regulating CCN1-Mediated AKT/NF-κB Signaling Pathway

**Figure 5:**
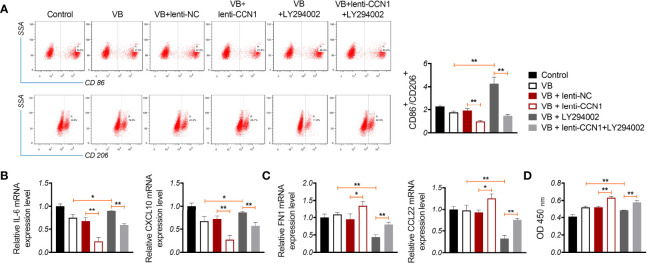
VB promotes M1 polarization by affecting CCN1-mediated AKT/NF-κB pathway. **(A)** The CD86^+^ and CD206^+^ ratio in macrophages were measured by flow cytometry. **(B, C)** The relative expression levels of IL6, CXCL10, FN1, and CCL22 were examined by RT-qPCR. **(D)** The proliferation of A2780 cells was detected by CCK8 assay. A2780 cells were co-cultured with THP-1 macrophage and treated with LPS + IFN-γ, 20 µM VB, lenti-NC, lenti-CCN1, or/and LY294002. Data are expressed as the mean ± SD; ^*^ *P* < 0.05 and ^**^ *P* < 0.01.

We verified whether VB promoted M1 polarization by blocking the CCN1-mediated AKT/NF-κB pathway. As shown in **Figures 5A, B**, CCN1 weakens the effect of VB on promoting M1 polarization, as evidenced by the increased CD86^+^/CD206^+^ ratio and expression of IL-6 and CXCL10 (*P* < 0.01). Contrarily, LY294002 potentiated the promotive effect of VB on M1 polarization. CCN1 overexpression also reversed the synergistic effect of VB and LY294002 on M1 polarization (**Figures 5A, B**). Moreover, CCN1 reversed the inhibitory effect of VB on M2 polarization, while LY294002 promoted this inhibitory effect of VB. CCN1 overexpression weakened the synergistic effect of VB and LY294002 on M2 polarization inhibition (**Figure 5C**). Subsequently, co-culturing macrophages with A2780 cells found that CCN1 overexpression enhanced the proliferation ability of A2780 cells, whereas LY294002 presented the opposite effect (*P* < 0.05; **Figure 5D**).

### VB Retards Tumour Growth *In Vivo* Through Suppressing CCN1-AKT/NF-κB Pathway and Promoting M1 Polarization

**Figure 6:**
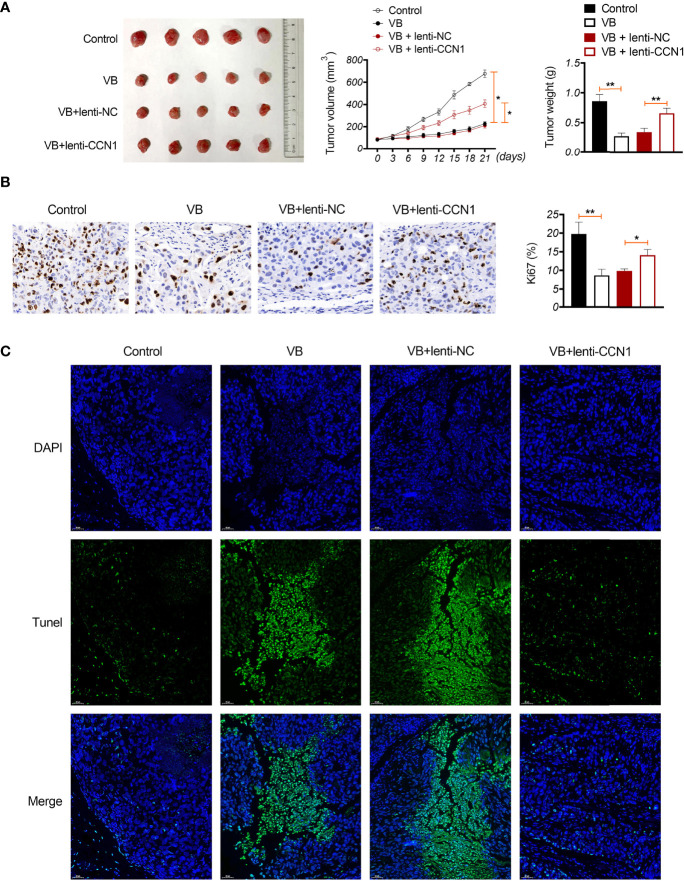
VB inhibits tumour formation *in vivo* in OC. **(A)** A2780 cells were subcutaneously injected into the right flank of nude mice to establish a tumour xenograft model. The tumour volume and weight were recorded. **(B)** Ki-67 expression in OC tissues. Scar bar = 50 µm. **(C)** Apoptosis was identified using TUNEL assay. Scar bar = 50 µm. Error bars represent mean ± SD; ^*^ *P* < 0.05 and ^**^ *P* < 0.01.

**Figure 7:**
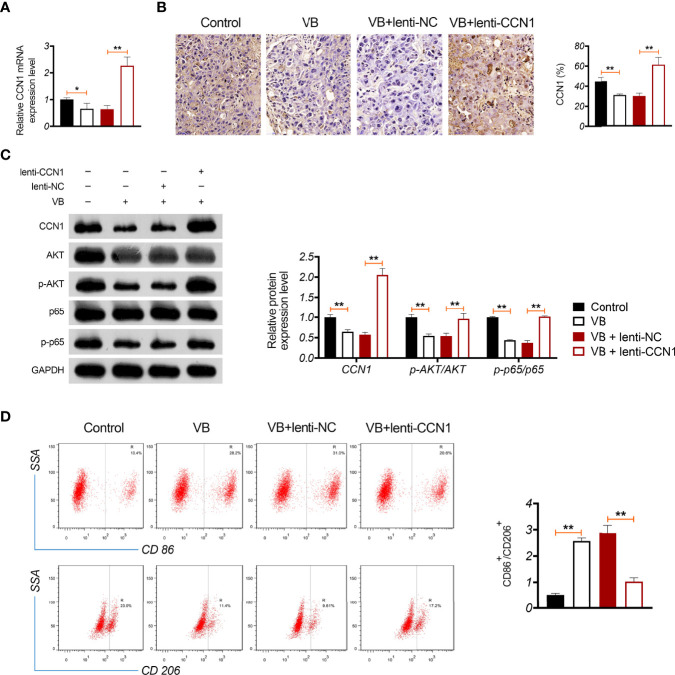
VB promotes M1 polarization in OC tissues through inhibiting CCN1-mediated AKT/NF-κB pathway. **(A)** The relative mRNA expression of CCN1 in tumour tissue was detected by RT-qPCR. **(B)** The CCN1 protein expression was detected in tumour tissue by immunohistochemistry. **(C)** The protein expression levels of CCN1, p-AKT/AKT, and p-p65/p65 were detected by western blotting in tumour tissue. **(D)** The ratio of CD86^+^/CD206^+^ in tumour tissues was measured by flow cytometry. Tumour tissue was removed from tumour xenograft model mice subcutaneously injected with A2780 cells. Each treatment was replicated three times. Error bars represent mean ± SD; ^*^ *P* < 0.05and ^**^ *P* < 0.01.

A xenograft model was established to evaluate the anti-tumour effects of VB *in vivo*. The results showed that VB significantly reduced the tumour volume and weight compared with that of the control (*P* < 0.05; **Figure 6A**). VB also downregulated Ki-67 expression (a cell proliferation marker) and induced apoptosis in tumour tissues compared to that in the control (**Figures 6B, C**). However, CCN1 overexpression weakened the inhibitory effect of VB on tumour growth (**Figures 6A–C**). We further confirmed the mechanism of action of VB involving CCN1-AKT/NF-κB pathway *in vivo*. Our results showed that VB significantly decreased the expression of CCN1, p-AKT, and p-p65 in OC tissues compared with that of the control (*P* < 0.05). CCN1 overexpression reversed the effect of VB on downregulating the expression of CCN1, p-AKT, and p-p65 (*P* < 0.01; **Figures 7A–C**). Moreover, VB treatment increased the ratio of CD86^+^/CD206^+^ cells compared with that of the control (*P* < 0.01). CCN1 overexpression also reduced the effect of VB on promoting M1 polarization (*P* < 0.001; **Figure 7D**).

## Discussion

OC causes high mortality among all gynaecological malignancies worldwide (1). Currently, TCM is a promising alternative adjuvant to traditional chemotherapy, which is advantageous for the prevention and treatment of OC (46, 47). VB is a bioactive constituent of diverse Chinese herbs that possess anti-cancer activities (48). In this study, we found that VB inhibited the proliferation and migration, and induced the apoptosis of OC cells. Meanwhile, VB induced polarization of macrophages into the M1 phenotype. Moreover, we clarified the mechanism by which VB induces M1 polarization, which is regulated by the CCN1-mediated AKT/NF-κB signalling pathway.

VB is a naturally occurring secondary metabolite widely existed in the plant kingdom, and possesses anti-tumour activity for the treatment of various cancers, such as human oral squamous cell carcinoma, colorectal cancer, and glioblastoma (33, 48–50). Zhang et al. demonstrated that VB effectively inhibited the growth and metastasis of human oral squamous cell carcinoma cells (49). VB also has anti-metastatic and anti-invasion activities in colorectal cancer (33). In our study, we found that VB inhibited cell proliferation and migration, and promoted apoptosis of OC cells. This result indicates that VB exerts an anti-tumour effect in OC.

Macrophages are an important cellular component in tumour tissues and are commonly referred to as tumour-associated macrophages (51). Tumour-associated macrophages can be polarized into two phenotypes, M1- and M2-like macrophages (52). A review has shown that M1 macrophage polarization exhibits anti-tumour effects and M2 polarization possesses pro-tumoral effects (53). Travers et al. suggested that M1 macrophage polarization ameliorates tumour burden and prolongs survival in an OC mouse model (54). Additionally, a previous study indicated that VB can inhibit the expression of pro-inflammatory factors induced by LPS (55). Zhang et al. demonstrated that VB possesses the inhibitory effect on macrophage infiltration (31). In our study, we found that VB increased the CD86^+^/CD206^+^ ratio (M1/M2 ratio) and upregulated the expression of IL-6 and CXCL10 (M1 biomarkers). VB-induced M1 polarization inhibited OC cell proliferation. These findings indicate that VB can induce M1 polarization, thereby suppressing OC cell growth.

CCN1 plays an essential role in tumorigenesis and inflammatory responses in various cancers, including OC (21, 56). For instance, CCN1 as a pro-inflammatory factor may promote tumor metastasis and progression in epithelial OC (21). The increase of CCN1 expression contributes to the proliferation of OC cells (57). In this study, molecular docking revealed that the chemical structure of VB was bound to eight amino acid sites (namely ASP-224, ARG-362, TRP-331, VAL-330, TYR-367, ARG-201, ASN-102, and SER-225) of the CCN1 protein. These results indicate that VB directly regulates CCN1 expression. In condition, the AKT/NF-κB signalling pathway plays a critical regulatory role in cancers, including OC (28, 58, 59). A previous study indicated that CCN1 promotes tumorigenicity by activating the AKT/NF-κB pathway in pancreatic cancer (60). In this study, we found that VB downregulated CCN1 and inhibited the AKT/NF-κB signalling pathway in OC cells. CCN1 overexpression weakened the inhibitory effect of VB on the AKT/NF-κB pathway, and the synergistic effect of VB and LY294002 (an AKT inhibitor). These results indicate that VB inhibits the CCN1-mediated AKT/NF-κB pathway in OC. Our further experiments exhibited that CCN1 overexpression weakened the anti-tumour effect of VB. CCN1 overexpression also reversed the combined effects of VB and LY294002 in suppressing the OC cell malignant progression. Meanwhile, we found that CCN1 knockdown enhanced the inhibitory effects of VB on OC cell viability, migration, and invasion (**Supplementary Figures 2A–C**). CCN1 knockdown also potentiated the effect of VB on restraining AKT/NF-κB pathway (**Supplementary Figure 2D**). These results demonstrate that VB restrains the progression and development of OC by retarding the CCN1-mediated AKT/NF-κB pathway.

The AKT/NF-κB signalling pathway is also a pivotal mediator of macrophage polarization (61). Wang et al. indicated that AKT/NF-κB signalling pathway promotes the polarization of macrophages to the M2 phenotype in pancreatic cancer (29). Le et al. reported that inhibition of the AKT/NF-κB pathway can restrain M2 macrophage polarization in OC (28). We considered that the effect of VB on inducing M1 polarization may also be associated with the CCN1-mediated AKT/NF-κB pathway. Our study showed that the promotive effect of VB on M1 polarization was weakened by CCN1 overexpression, whereas it was potentiated by LY294002. CCN1 overexpression reversed the combined effect of VB and LY294002 on macrophage polarization to the M1 phenotype. These results further indicate that VB promotes M1 polarization by affecting the CCN1-mediated AKT/NF-κB pathway in OC.

Furthermore, a xenograft model was established to verify the inhibitory effect of VB on OC and its related mechanisms *in vivo*. We identified that VB reduced tumour volume and weight, inhibited cell proliferation, and promoted apoptosis in OC tissues. However, CCN1 overexpression weakened the effect of VB on retarding OC growth *in vivo*. This suggests that VB exerts an anti-tumour effect in OC *in vivo* by downregulating CCN1. In addition, CCN1 overexpression reversed the effect of VB on suppressing the CCN1-mediated AKT/NF-κB pathway in OC *in vivo*. This finding suggests that VB can inhibit the CCN1-mediated AKT/NF-κB pathway in OC tissues. Subsequently, we found that CCN1 weakened the promoting effect of VB on M1 polarization. This result further illustrates that VB can promote M1 polarization by downregulating CCN1 in OC tissues.

## Conclusion

VB is a potential therapeutic drug for OC treatment. The anti-tumour effect of VB was associated with the activation of M1 polarization regulated by the CCN1-mediated AKT/NF-κB pathway. These findings provide a promising drug for OC treatment and elucidate the underlying mechanism of VB against OC. However, the regulatory association between the CCN1-AKT/NF-κB pathway and the macrophage polarization has not been fully illustrated, which will be further investigated in our subsequent work. Clinical trials are also required to verify the efficacy of VB in OC treatment. The specific mechanism by which VB affects macrophage polarization has not been elucidated in detail.

## Data Availability Statement

The original contributions presented in the study are included in the article/**Supplementary Material**. Further inquiries can be directed to the corresponding author.

## Ethics Statement

The animal study was reviewed and approved by the Animal and Medical Committee of Inner Mongolia People’s Hospital.

## Author Contributions

YR performed the conception, design of the research, statistical analysis, obtained the funding and draft the manuscript. WZ and JH acquired and analysed data. YM revised the manuscript. All authors contributed to the article and approved the submitted version.

## Funding

This work was supported by the Natural Science Foundation of Inner Mongolia Autonomous Region [No. 2020MS08154] and the Health Science and Technology Foundation of Inner Mongolia Autonomous Region [No.202202005].

## Conflict of Interest

The authors declare that the research was conducted in the absence of any commercial or financial relationships that could be construed as a potential conflict of interest.

## Publisher’s Note

All claims expressed in this article are solely those of the authors and do not necessarily represent those of their affiliated organizations, or those of the publisher, the editors and the reviewers. Any product that may be evaluated in this article, or claim that may be made by its manufacturer, is not guaranteed or endorsed by the publisher.

## Supplementary Material

Supplementary Figure 1 The IC50 values of VB treating A2780 and SKOV3 cells at 48 h were calculated based on the CCK8 assay.

Supplementary Figure 2 VB inhibits the malignant characteristics of OC cells *via* deactivating the CCN1-mediated AKT/NF-κB pathway. **(A)** The viability of A2780 and SKOV3 cells was examined using the CCK8 assay. **(B, C)** The migration and invasion of A2780 and SKOV3 cells was measured by transwell assay. Scar bar = 50 µm. **(D)** The protein levels of p-AKT/AKT and p-p65/p65 were detected by western blotting in A2780 and SKOV3 cells. A2780 and SKOV3 cells were treated with si-CCN1, lenti-CCN1, or/and 20 µM VB. Each treatment was replicated three times. Error bars represent mean ± SD; ^**^ *P* < 0.01 vs. the si-CCN1 group; ^##^ *P* < 0.01 vs. the lenti-CCN1 group.
