# Supplementary figures and images for: The Anti-Tumor Efficacy of Verbascoside on Ovarian Cancer via Facilitating CCN1-AKT/NF-κB Pathway-Mediated M1 Macrophage Polarization
Source: Front Oncol. 2022 Jun 17;12:901922. doi: 10.3389/fonc.2022.901922 (PMC9249354; doi:10.3389/fonc.2022.901922)

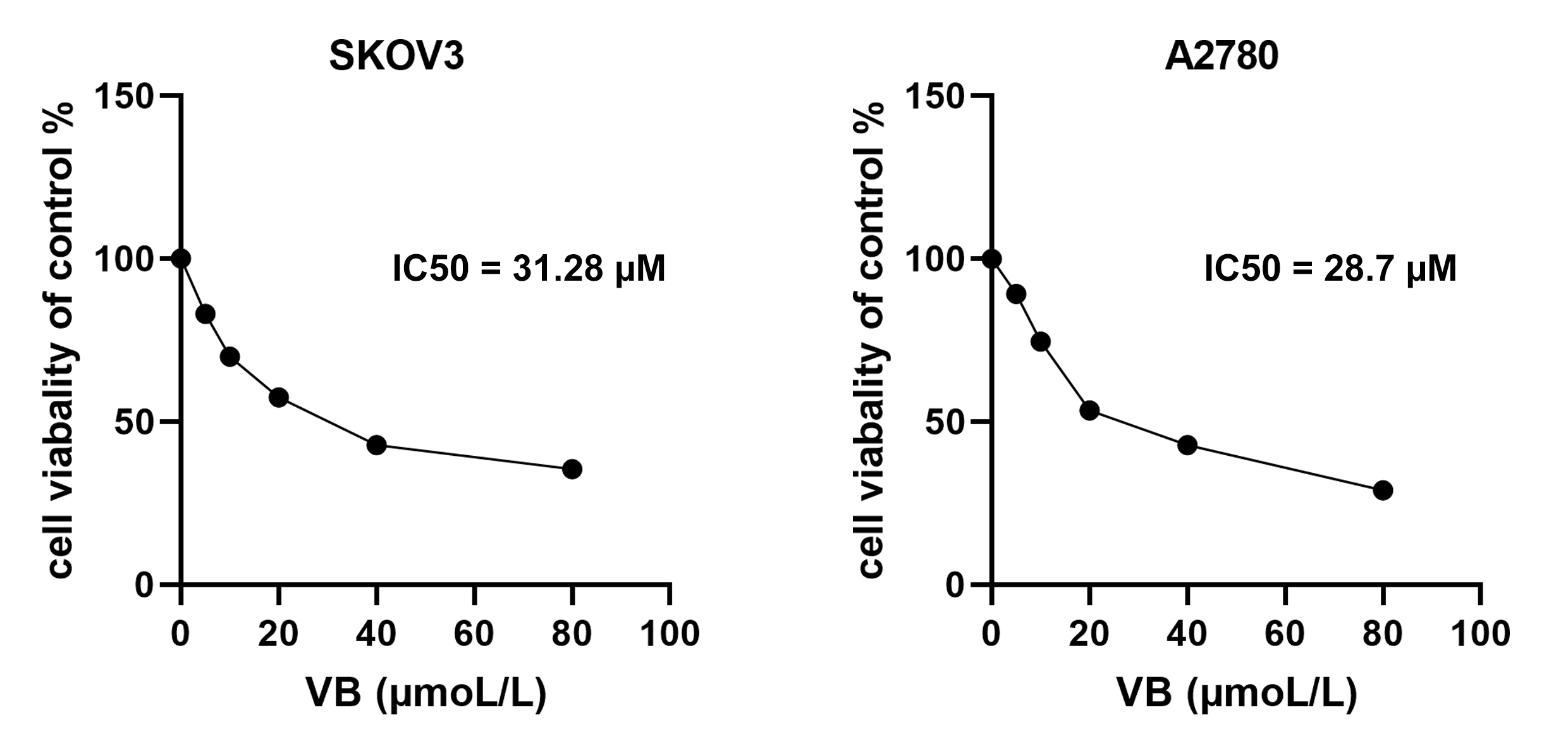

Supplement: Supplementary Figure 1 — The IC50 values of VB treating A2780 and SKOV3 cells at 48 h were calculated based on the CCK8 assay. [file Image_1.tif]

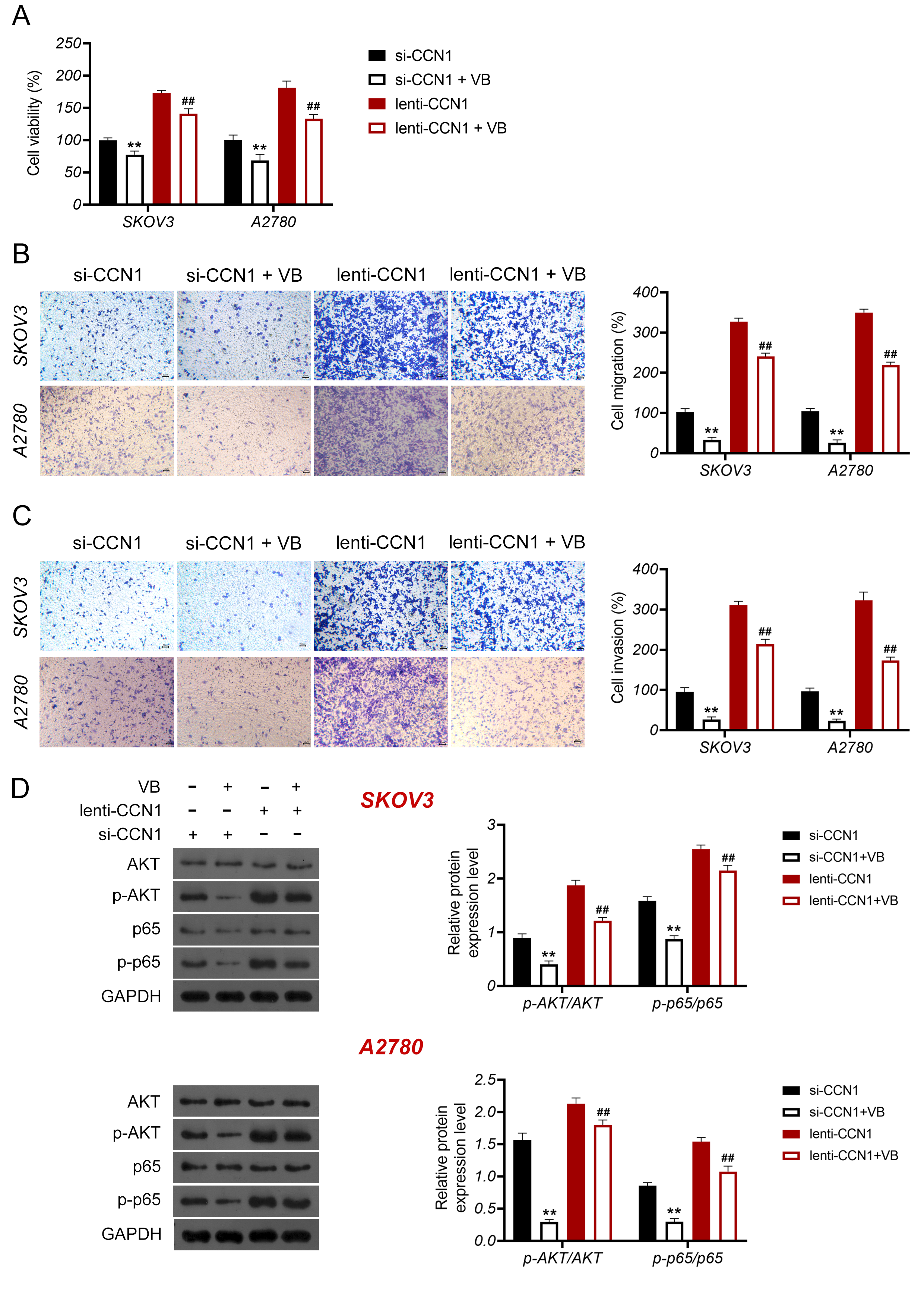

Supplement: Supplementary Figure 2 — VB inhibits the malignant characteristics of OC cells via deactivating the CCN1-mediated AKT/NF-κB pathway. (A) The viability of A2780 and SKOV3 cells was examined using the CCK8 assay. (B, C) The migration and invasion of A2780 and SKOV3 cells was measured by transwell assay. Scar bar = 50 µm. (D) The protein levels of p-AKT/AKT and p-p65/p65 were detected by western blotting in A2780 and SKOV3 cells. A2780 and SKOV3 cells were treated with si-CCN1, lenti-CCN1, or/and 20 µM VB. Each treatment was replicated three times. Error bars represent mean ± SD; ** P < 0.01 vs. the si-CCN1 group; ## P < 0.01 vs. the lenti-CCN1 group. [file Image_2.tif]
